# Influence of *Salmonella enterica* serovar Enteritidis infection on the composition of chicken cecal microbiota

**DOI:** 10.1186/1746-6148-9-140

**Published:** 2013-07-15

**Authors:** Petra Videnska, Frantisek Sisak, Hana Havlickova, Marcela Faldynova, Ivan Rychlik

**Affiliations:** 1 Veterinary Research Institute, Brno, Czech Republic

**Keywords:** Chicken, Microbiome, Intestinal tract, Pyrosequencing, Salmonella

## Abstract

**Background:**

Infection of newly hatched chicks with *Salmonella enterica* serovar Enteritidis (*S*. Enteritidis) results in an inflammatory response in the intestinal tract which may influence the composition of gut microbiota. In this study we were therefore interested whether *S*. Enteritidis induced inflammation results in changes in the cecal microbiota. To reach this aim, we compared the cecal microbiota of non-infected chickens and those infected by *S*. Enteritidis by pyrosequencing the V3/V4 variable regions of genes coding for 16S rRNA.

**Results:**

Cecal microbiota of chickens up to 19 days of life was dominated by representatives of *Enterobacteriaceae*, *Lachnospiraceae* and *Ruminococcaceae*, followed by *Lactobacillaceae*. The presence of *Lachnospiraceae* did not change after *S*. Enteritidis infection. *Enterobacteriaceae* increased and *Ruminococcaceae* decreased after *S*. Enteritidis infection in two independent experiments although these results were not significant. A significant increase in both experiments was observed only for the representatives of *Lactobacillaceae* which may correlate with their microaerophilic growth characteristic compared to the obligate anaerobes from the families *Lachnospiraceae* and *Ruminococcaceae*.

**Conclusions:**

We conclude that *S*. Enteritidis infection influences the composition of the cecal microbiota in chickens but these changes are minor in nature and should be understood more as an indirect consequence of infection and inflammation rather than a positively selected evolutionary trait.

## Background

Gut microbiota plays an important role in shaping the host’s immune response, nutrient uptake and production of metabolites essential for the host [1-4]. The composition of gut microbiota is not constant and develops over time. In warm blooded vertebrates, the initial gut colonizers are those belonging to the phylum *Proteobacteria*, particularly the family *Enterobacteriaceae*[5,6]. Later in life, representatives from the phylum *Firmicutes* consisting of the families *Lachnospiraceae*, *Ruminococcaceae*, *Clostridiaceae* or *Lactobacillaceae* dominate and in older animals, the digestive tract becomes populated with representatives from the phylum *Bacteroidetes*[5-7]. Besides the above mentioned life stage factor, the composition of gut microbiota is shaped by food or feed composition and, of course, is highly responsive to extreme interventions like antibiotic therapy [8-11].

The composition of gut microbiota may also change during various diseases which are associated with inflammation and an influx of phagocytes and lymphocytes from the circulation [12-14]. Changes in the composition of gut microbiota caused by inflammation are observed also in animals or humans infected with non-typhoid serovars of *Salmonella enterica*[15-18]. An influx of phagocytes into the inflamed intestinal tract following *S*. *enterica* infection results in the production of antimicrobial metabolites such as proteases, reactive oxygen species, nitric oxide radicals and chelators of bacterial siderophores. Some of these antimicrobial products result not only in pathogen inactivation but also in damage to the host’s own tissue, particularly damage to the integrity of the intestinal epithelium and efflux of electrolytes clinically manifesting as diarrhea. It is therefore legitimate to hypothesize that the pathogen inducing the inflammation should be adapted to life in such an environment and the ability to survive in inflamed tissue is considered as an evolutionary adaptation also in *Salmonella*[19]. Lipocalin 2 produced by murine neutrophils in the intestine of streptomycin-treated mice in response to *S*. *enterica* infection binds to enterochelin, a type of bacterial siderophore. However, *S*. *enterica* produces glycosylated forms of enterochelin and this type of siderophore is not captured by lipocalin 2. In this way, *S*. *enterica* gets a growth advantage in the inflamed intestine over the microbiota members expressing the non-glycosylated forms of enterochelins [20]. An additional advantage *S*. *enterica* has over the other microbiota members is its ability to respire tetrathionate. Although this electron acceptor is absent from a normal, healthy intestinal tract, it is produced in the inflamed intestine from common reduced sulfur compounds by oxidative species produced by infiltrating neutrophils [21]. However, it is unknown to what extent these *S*. *enterica* adaptations will result in changes to the composition of gut microbiota.

Infection of newly hatched chicks with *Salmonella enterica* serovars Enteritidis or Typhimurium (*S*. Enteritidis or *S*. Typhimurium) results in an inflammatory response in the intestinal tract [22-24]. We were therefore interested whether the inflammation induced by *Salmonella* infection will or will not affect the development and composition of gut microbiota in chickens. Our previous results using T-RFLP and quantitative real time PCR indicated that the changes in gut microbiota in chickens are not as dramatic as one would expect from data in other models of host - pathogen interactions [12-14,16]. In this study we have therefore used pyrosequencing of V3 and V4 variable regions of 16S rRNA genes to characterize the consequences of *S*. Enteritidis infection on the composition of chicken cecal microbiota in detail. Although we identified species which decreased or increased after the infection, overall we did not detect any large scale changes indicating that modification and overgrowth of the cecal microbiota is not a major driving force in the evolution of *Salmonella* – chicken interactions.

## Results

**Figure 1:**
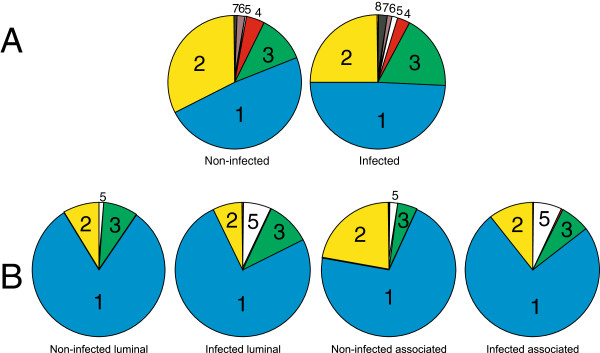
**Composition of chicken microbiota with or without of *S. *Enteritidis infection. **The cecal microbiota in young chickens were dominated by representatives of the family *Lachnospiraceae *(phylum *Firmicutes*) followed by *Ruminococcaceae *(*Firmicutes*) and *Enterobacteriaceae* (*Proteobacteria*). Infection of chickens with *S*. Enteritidis repeatedly increased representatives of *Lactobacillaceae*. Panel **A**, experiment 1, for which the figures were generated by averaging the microbiota composition of all time points i.e. from day 7, 10, 13, 16 and 19. Panel **B **- experiment 2, in which the luminal and mucus associated microbiota were characterized separately. 1 – *Lachnospiraceae*, 2 – *Ruminococcaceae*, 3 – *Enterobacteriaceae*, 4 – unclassified *Clostridiales*, 5 – *Lactobacillaceae*, 6 – *Catabacteriaceae*, 7 – *Clostridiaceae*, 8 - *Bifidobacteriaceae*.

Altogether, 362 609 reads were analyzed in this study following pyrosequencing the amplification products of the V3/V4 hypervariable 16S rRNA genes. These reads were distributed among 2079 different OTUs (Operational Taxonomic Unit) identified in 30 samples from the non-infected chickens and 20 samples from *S*. Enteritidis infected chickens (we failed with pyrosequencing of one sample originating from a 19-day-old chicken infected with *S*. Enteritidis on day 16). Number of reads per sample ranged from 1,141 to 21,109 (see Table 1 and Additional file 1). The composition of cecal microbiota was similar to that reported previously for young chickens [5,7,16,25]. The cecal microbiota in young chickens was dominated by representatives of the family *Lachnospiraceae* (phylum *Firmicutes*) followed by *Ruminococcaceae* (*Firmicutes*) and *Enterobacteriaceae* (*Proteobacteria*). The presence of the predominant family *Lachnospiraceae* was unaffected by *S*. Enteritidis infection. Infection of chickens with *S*. Enteritidis (see Table 2 for *S*. Enteritidis counts) caused a minor numerical increase in *Enterobacteriaceae* in the microbiota of infected chickens at the expense of *Ruminococcaceae* in both experiments. In addition, although forming a minority population in the cecum, representatives of *Lactobacillaceae* repeatedly increased after the infection with *S*. Enteritidis (Figure 1).

**Table 1:** Basic characteristics of chicken cecal microbiota samples for individual time points shown as average ± SD out of 3 independent chickens

**Experiment 1**	NI4*	**NI7**	**NI10**	**NI13**	**NI16**	**NI19**
No. reads	2409±265	4198±1570	3314±1097	7577±455	14994±2354	9586±2045
Observed species	54±6	107±18	94±19	153±33	307±42	233±28
Chao1 estimate	69±8	199±81	146±40	276±88	605±103	418±79
Shannon index	3.15±0.33	4.33±0.20	4.54±0.02	4.78±0.26	5.39±0.22	5.42±0.11
Simpson index	0.82±0.04	0.91±0.01	0.94±0.00	0.93±0.02	0.96±0.00	0.96±0.00
**Experiment 1**		**S7**	**S10**	**S13**	**S16**	**S19**
No. reads		9940±3942	16514±2740	12725±3783	5840±921	17992±3235
Observed species		155±81	187±8	196±47	149±5	479±11
Chao1 estimate		277±98	298±66	289±76	265±36	846±80
Shannon index		4.15±0.36	4.61±0.18	4.53±0.39	5.05±0.37	5.55±0.14
Simpson index		0.89±0.02	0.92±0.02	0.92±0.03	0.94±0.02	0.96±0.00
**Experiment 2**	**Lumen N7**	**Lumen N10**	**Lumen S10**	**Mucus NI7**	**Mucus NI10**	**Mucus S10**
No. reads	3302±391	6958±4142	4107±3942	1307±161	4278±79	1826±404
Observed species	184±33	221±24	196±81	95±5	132±15	87±10
Chao1 estimate	283±76	319±42	298±98	150±21	204±26	110±2
Shannon index	5.24±0.31	4.98±0.43	5.00±0.36	4.82±0.07	4.64±0.08	4.63±0.26
Simpson index	0.95±0.01	0.93±0.03	0.93±0.02	0.93±0.01	0.93±0.00	0.92±0.02

* NI - non-infected chicken followed by age of chickens in days; S - *Salmonella* Enteritidis infected chickens followed by age of chickens in days, Lumen and Mucus - luminal and mucus associated microbiota characterized separately in experiment 2.

**Table 2:** ***S***. **Enteritidis colonization of the caecum of chickens after oral infection**

	**log CFU/g of caecum**	
Age (days)*	**Exp1**	**Exp2**
7	7.96±0.35	ND
10	7.96±0.22	6.73±0.06
13	8.13±0.08	ND
16	7.57±0.49	ND
19	7.44±0.82	ND

*age of chickens means age when the chickens were sacrificed, i.e. the chickens were therefore infected 3 days earlier. *ND* not done.

**Figure 2:**
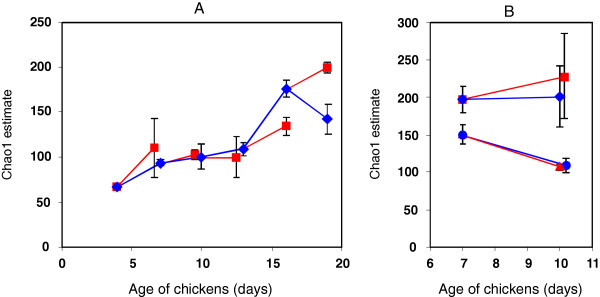
**Influence of *S*. Enteritidis on the estimated number of OTUs. **Chickens were infected with *S*. Enteritidis at 3-day intervals and sacrificed 3 days later (red lines) with appropriate non-infected control chickens (blue lines). No reproducible effect of *S*. Enteritidis infection on total OTU numbers was recorded. Panel **A**, experiment 1. Panel **B**, experiment 2 in which luminal microbiota diamonds and squares) and mucus-associated microbiota (triangles and circles) were characterized independently. Data are presented as averages from 3 chickens sacrificed at each time point ± standard deviation. In days 7, 10 and 13 in Panel **A**, and day 10 in Panel **B**, the two samples are slightly shifted to avoid overlaying standard deviations one over another.

In the next analysis we compared whether *S*. Enteritidis infection affected the total complexity of chicken cecal microbiota characterized by the number of different OTUs predicted as Chao1 estimates. Rather unexpectedly, infection of 4, 7, 10, 13 and 16-day-old chickens with *S*. Enteritidis did not result in reproducible changes in the number of OTUs in the cecum. This was confirmed also in the repeated experiment in which luminal and mucus-associated microbiota were characterized separately (Figure 2).

**Figure 3:**
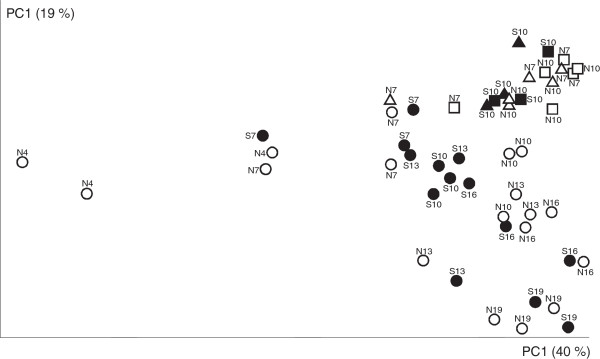
**PCoA of infected and non**-**infected chickens.** Circles, individual chickens and their cecal microbiota from experiment 1. Squares, chickens and their mucosa-associated microbiota from experiment 2. Triangles, chickens and their luminal microbiota from experiment 2. Black symbols - infected chickens, white symbols – non-infected chickens. Numbers indicate age of individual chickens.

Since the total number of OTUs may not properly characterize the microbial populations in the cecum of *S*. Enteritidis infected or non-infected chickens, the microbiota of individual chickens was compared further by UniFrac analysis followed by PCoA. PCoA clustered chicken microbiota from the two experiments into separate clusters. However, no obvious clustering of the infected and non-infected chickens was recorded in either of the experiments (Figure 3).

**Figure 4:**
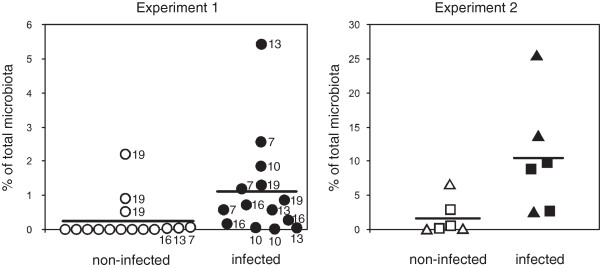
**Relative representation of *Lactobacillus ultunensis *(experiment 1) and *Lactobacillus gasseri *(experiment 2) in the chicken cecum with or without *S. *Enteritidis infection. **Circles, percentage of *L*. *ultunensis* out of the total microbiota in individual chickens in experiment 1. Squares, percentage of *L*. *gasseri *from the mucus-associated microbiota in the cecum of individual chickens in experiment 2. Triangles, percentage of *L*. *gasseri* from the luminal microbiota in the cecum of individual chickens in experiment 2. Black symbols - infected chickens, white symbols – non-infected chickens. Numbers, indicate the age of individual chickens. Age of the chickens is not shown for *L*. *ultunensis* negative chickens in experiment 1, and chickens in experiment 2 as all these chickens were ten days old. The horizontal line in each experiment represents the mean of all chickens. Comparisons of *Lactobacilli* prevalence in both the experiments by t-test came out as significantly different at p<0.05.

The contradictory results presented in Figures 1, 2 and 3, could be influenced by the fact that representatives of the predominant family *Lachnospiraceae* were not affected by *S*. Enteritidis thus masking the effect of *S*. Enteritidis infection on the minority populations. Finally we therefore analyzed individually all 2079 different OTUs for significant changes in response to *S*. Enteritidis infection by t-test considering the comparisons with p<0.05 as significant. In experiment 1, none of the OTUs significantly decreased after *S*. Enteritidis infection. On the other hand, six OTUs significantly increased after *S*. Enteritidis infection. Three of them belonged to the family *Enterobacteriaceae* (one of them being *Salmonella*), and the remaining ones belonged to the families *Lachnospiraceae*, *Lactobacillaceae* and *Bifidobacteriaceae*. Five of these OTUs formed less than 0.02% of the total microbiota in the non-infected chickens and still less than 0.1% after infection. The only OTU which significantly increased after the infection forming around 1% of total microbiota was the representative of *Lactobacillaceae*. This OTU formed 0.24% of the total microbiota in the cecum of non-infected chickens and increased to 1.10% after infection with *S*. Enteritidis (Figure 4). RDP seqmatch analysis showed that the rRNA sequence of this OTU was identical to *Lactobacillus ultunensis*.

In experiment 2, six OTUs significantly decreased and 2 OTUs significantly increased after *S*. Enteritidis infection. Five of the OTUs that decreased after infection belonged to the family *Lachnospiraceae* and the remaining one belonged to the family *Streptococcaceae*. Two OTUs which significantly increased after *S*. Enteritidis infection belonged to families *Lactobacillaceae* and *Ruminococcaceae*. As in experiment 1, only the representative of *Lactobacillaceae* formed more than 1% of the total microbiota, specifically 1.70% in the cecal microbiota of the non-infected chickens and 10.44% after *S*. Enteritidis infection (Figure 4). RDP seqmatch analysis showed that the rRNA sequence of this OTU was identical to *Lactobacillus gasseri*.

## Discussion

Chicken gut microbiota is understood to be one of the most important components of the host’s resistance to *Salmonella* infection as older chickens are more resistant to *Salmonella* infection than the younger ones [26,27] and the protective effect of preparations containing gut microbiota from healthy hens has been described [28-31]. In addition, antibiotic therapy in chickens which considerably affects gut microbiota [11] makes the chickens more susceptible to *Salmonella* infection [32-34]. This clearly points towards the role of the gut microbiota in host resistance and its influence on the maturation of the host immune system. However, it is unclear whether *Salmonella* infection leads to changes in the microbiota composition resulting in a growth advantage for *Salmonella*.

In this study we therefore addressed this topic using a model of *S*. Enteritidis and newly hatched chicks. Unlike our expectations but similar to previous reports [16,35], the changes in cecal microbiota after *S*. Enteritidis infection were quite low. The predominant microbiota remained unaffected by the infection as documented by minimal differences in the total number of OTUs before and after the infection. Similarly, no obvious clustering was recorded after PCoA analysis. Even a separate analysis of luminal and mucus-associated microbiota did not show any clear profile although the changes were expected to be more pronounced in mucus-associated microbiota due to the inflammation induced by *Salmonella* infection and the change in redox status close to the mucosa [21].

Similar to our previous study [16] we noticed that *Enterobacteriaceae* increased and *Ruminococcaceae* decreased in both experiments, despite not reaching statistical significance. The only significant difference was the increase of *Lactobacillaceae* after *S*. Enteritidis infection. This was rather unexpected, however, we believe that this observation is correct for the following reasons. First, a significant increase in *Lactobacillaceae* was observed in both experiments. Second, even though *Lactobacillaceae* was not the predominant family in the chicken cecum, this family still formed around % of the total microbiota and its changes were therefore not observed in the minority population which might be subjected to a greater sample-to-sample variation. Third, the increase in *Lactobacillaceae* was caused by an increase in the major OTUs of this family in both experiments. This was different from some of the OTUs of *Lachnospiraceae* or *Ruminococcaceae* families which showed a significant difference in one of the two experiments but such OTUs always represented a minority of the OTUs within these families. Fourth, the two OTUs of *Lactobacillaceae* which increased in experiments 1 and 2 belonged to two different species, *Lactobacillus ultunensis* and *Lactobacillus gasseri*, respectively. The increase of *Lactobacillaceae* after *S*. Enteritidis infection was therefore not specific for a particular *Lactobacillus* clone but seems to be general, irrespective of *Lactobacillus* species. Finally, unlike members of *Ruminococcaceae* or *Lachnospiraceae* which are obligate anaerobes, *Lactobacilli* are microaerophilic bacteria which may allow them to survive under conditions with increased redox potential due to the production of reactive oxygen species by granulocytes infiltrating the site of inflammation [15,21,36].

## Conclusions

Based on our results we conclude that *S*. Enteritidis infection in young chickens influences the microbiota composition, however, the scope of these modifications is minor. Changes in chicken cecal microbiota after *S*. Enteritidis infection can be therefore characterized more as an indirect consequence of the infection rather than a positively selected evolutionary trait.

## Methods

### Experimental animals and sample collection

Male ISA Brown chickens (Hendrix Genetics, Boxmeer, The Netherlands) were obtained from a local commercial hatchery on the day of hatching. The parents of the chickens used in this study were vaccinated against salmonellosis. The chickens were reared in wire cages in the experimental animal house and allowed free access to water and pathogen, antibiotic and coccidiostatic-free feed. Three chickens were sacrificed on day 4, 7, 10, 13, 16 and 19 of life. In addition, 3 chickens were orally infected with *S*. Enteritidis 147 of phage type 4 [36] when aged 4, 7, 10, 13 and 16 days and were sacrificed 3 days post-infection. After the inoculation, the orally infected chickens were housed separately from the non-infected controls. Short segments of cecum together with its contents were collected from both the infected and non-infected chickens and were frozen at -20°C within 10 min after collection.

In the second experiment we sacrificed 7- and 10-day-old chickens, three chickens for each time point. In addition, a group of 3 chickens was orally infected with *S*. Enteritidis when aged 7 days and sacrificed 3 days later. During post mortem sample collection, the cecal contents were squeezed out of the cecum and collected separately. The cecal wall was then washed 3 times in PBS with gentle shaking to remove all luminal bacteria and mucus associated bacteria were collected from the washed cecum by scraping the mucus with a plastic scapula. The cecal contents and mucus-associated microbiota samples were then frozen at -20°C within 10 min and saved for no longer than 2 months until DNA purification.

All animal treatments and handling have been performed according to current Czech legislation and have been approved by the Ethics Committee of the Czech Ministry of Agriculture.

### DNA purification

After slow defrosting at room temperature, approx. 240–260 mg of total cecal content from experiment 1, and the cecal contents or mucus samples from experiment 2 were homogenized for 1 min at 7000 RPM in the MagNALyzer (Roche Diagnostics) using zirconia silica beads (BioSpec Products). Following homogenization, the DNA was extracted using a QIAamp DNA Stool Mini Kit (Qiagen) according to the manufacturer’s instructions and purified DNA was stored at -20°C until use.

### Pyrosequencing

The purified DNA was used as a template in PCR with the forward primer 5' CGTATCGCCTCCCTCGCGCCATCAG – MID-*GGAGGCAGCAGTRRGGAAT* 3', and reverse primer 5' CTATGCGCCTTGCCAGCCCGCTCAG- MID- *CTACCRGGGTATCTAATCC* 3' using HotStarTaq Master Mix Kit following instructions of the manufacturer (Qiagen). The underlined sequences were required at different steps of pyrosequencing while those in italics are sequences complementary to the conserved parts of 16S rDNA flanking the V3/V4 hypervariable region [37]. The 454 Standard MID Set for sample barcoding was used. Cycling conditions consisted of hot start at 95°C for 15 min followed by 30 cycles of incubation at 94°C for 40 s, 55°C for 55 s and 72°C for 60 s. PCR was terminated by a final extension at 72°C for 5 min. After PCR, the amplification products approx. 525 bp in size were separated electrophoretically in a 1.2% agarose gel, gel-purified using a QIAquick Gel Extraction Kit (Qiagen) and subjected to pyrosequencing. Pyrosequencing was performed using GS Junior Titanium sequencing chemistry and a GS Junior 454 sequencer exactly according to the manufacturer’s instructions (Roche). In one sequencing run, the amplification products from 15 to 24 samples were mixed and analyzed.

### Sequence analysis

Fasta and qual files generated as an output of the pyrosequencing were uploaded into Qiime software [38]. Quality trimming criteria included no mismatch in MID sequences and a maximum of 1 mismatch in primer sequences. The obtained sequences with qual score higher than 20 were shortened to the same length of 350 bp and classified with RDP Seqmatch with an OTU discrimination level set to 97%. In the next step, chimeric sequences were predicted and excluded from the analysis. Diversity analyses (rarefaction curves and Chao1 richness) on OTU clusters were performed both using all sequences available for each sample and using the same number of randomly selected sequences adjusted to the number of sequences available for the sample with the lowest coverage. Finally, UniFrac analysis [39] followed by principal coordinate analysis (PCoA) was used to characterize the diversity in the microbial populations tested. The significance of increase or decrease of particular OTUs was calculated using a t-test comparing the percentage representation of each OTU in microbiomes of all infected and all non-infected chickens, separately for experiment 1 and experiment 2. For this analysis, the microbiomes of 4-day-old, non-infected chickens in experiment 1, and 7-day-old, non-infected chickens in experiment 2, were excluded from the analysis as these had no age-matched infected counterparts.

## Competing interests

The authors state that they do not have and financial or personal conflicts that could inappropriately bias their work.

## Authors’ contributions

PV performed the pyrosequencing, analysed the data and helped to draft the manuscript. MF purified the DNA. FS and HH were responsible for the animal experiments and sample collection. IR participated in the design of the study, data analysis and helped to draft the manuscript. All authors read and approved the final manuscript.

## Supplementary Material

Additional file 1 List of all OTUs identified in this study. Click here for file
